# Screening and prevention of gynecologic malignancies in patients with lynch syndrome: following the guidelines

**DOI:** 10.3389/fonc.2025.1563022

**Published:** 2025-03-12

**Authors:** Chen Ben David, Yoav Siegler, Revital Linder, Amnon Amit, Emad Matanes

**Affiliations:** ^1^ Department of Obstetrics and Gynecology, Rambam Health Care Campus, Haifa, Israel; ^2^ Ruth and Bruce Rappaport Faculty of Medicine, Technion, Haifa, Israel

**Keywords:** lynch syndrome, endometrial cancer, gynecologic cancer diagnosis, MIS, ovaria cancer

## Abstract

Lynch syndrome (LS), a hereditary condition caused by germline mutations in mismatch repair (MMR) genes, significantly elevates the lifetime risk of endometrial cancer (EC) (40–60%) and ovarian cancer (8–10%) in affected women. Despite advances in colorectal cancer screening for LS patients, optimal strategies for gynecologic cancer prevention remain under debate. Current recommendations for EC surveillance, including annual transvaginal ultrasound and endometrial biopsy starting at age 30–35, lack robust evidence for effectiveness. Risk-reducing hysterectomy with bilateral salpingo-oophorectomy (BSO) is frequently advised after childbearing to mitigate cancer risk. Emerging data suggest that hormonal interventions, such as oral contraceptives and progestin-based therapies, may reduce EC risk by up to 50%, offering non-surgical preventive options. Lifestyle modifications, including weight management and physical activity, further complement risk reduction strategies. Molecular diagnostic advancements, including immunohistochemistry and microsatellite instability testing, enhance early identification of LS-associated gynecologic malignancies. For patients with advanced or recurrent EC, the integration of immunotherapy into treatment regimens has demonstrated significant efficacy. Agents such as pembrolizumab and dostarlimab, particularly in combination with carboplatin and paclitaxel, have improved progression-free and overall survival rates for patients with MMR-deficient tumors. This review highlights the need for personalized, evidence-based approaches to gynecologic cancer screening and prevention in LS, emphasizing the importance of integrating genetic testing, patient education, and novel therapeutic options. Future research should focus on refining screening protocols and expanding non-invasive preventive strategies to improve outcomes for this high-risk population.

## 1 Introduction

Lynch syndrome (LS), previously known as hereditary non-polyposis colorectal cancer (HNPCC), is an autosomal dominant disorder caused by germline mutations in mismatch repair (MMR) genes. These mutations increase the risk of several cancers, with colorectal and endometrial cancers (EC) being the most prevalent. Women carrying one of the MMR genes mutations have a lifetime risk of EC ranging from 40% to 60%, making it the most common extracolonic cancer in this population (1, 2). Following that, the lifetime risk of ovarian cancer (OC) for women with LS is approximately 8-10% (3), significantly higher than that of the general population, 1.8% (4).

Given the high risk of gynecologic malignancies, early detection and preventive strategies are critical to improve outcomes in women with LS. Unlike colorectal cancer, which is routinely screened with colonoscopy or fecal occult blood test, no universal consensus exists for the screening of gynecologic cancers in LS. Current recommendations, including those from the National Comprehensive Cancer Network (NCCN), suggest that women with LS consider annual transvaginal ultrasound and endometrial biopsy starting at the age of 30 to 35 years, though evidence supporting the effectiveness of these methods remains limited (5). Total hysterectomy with bilateral salpingo-oophorectomy (BSO) is a risk-reducing strategy often recommended after childbearing is complete (6).

Hormonal therapies, including the use of oral contraceptives (OCPs) and progestin-based treatments have also been investigated as potential preventive measures for EC in this high-risk population (7).

The objective of this review is to critically evaluate the current strategies for the screening and prevention of gynecologic malignancies among patients with LS. We will explore the effectiveness of existing screening guidelines, review advancements in molecular diagnostics, and discuss the role of risk-reducing surgery and hormonal interventions.

## 2 Background on Lynch syndrome

LS is caused by germline mutations in the MMR genes, leading to defective DNA repair mechanisms and microsatellite instability (MSI). This impairment in the MMR system results in the accumulation of mutations in tumor suppressor genes and oncogenes resulting in accelerating carcinogenesis (8). This rapid accumulation of mutations due to MSI, explains the earlier onset of malignancies in women with LS compared to the general population (9).

The mutated genes in women with LS primarily include: MLH1, MSH2, MSH6, and PMS2 (10). In addition, germline promoter hypermethylation of MLH1 as an alternative genetic pathway (11). Additionally, deletions in the Epithelial cell adhesion molecule (EPCAM) gene, which is located upstream of MSH2, can lead to LS by causing epigenetic silencing of MSH2 (12).

The distribution of mutations among these genes is approximately:

- MLH1 and MSH2: 64% of mutations
- MSH6: 18% of mutations
- PMS2: 13% of mutations
- EPCAM: 3% of mutations (10).

EC is the most common extracolonic cancer in women with LS. The lifetime risk of developing endometrial cancer varies depending on the specific gene mutation:

- MLH1 and MSH2 mutations: 40-60%
- MSH6 mutations: 16-26%
- PMS2 mutations: 15 (13).

The median age of EC diagnosis in LS patients is approximately 50 years, about 10 years earlier than in the general population. In some LS families, particularly those with MSH6 mutations, the risk of EC may exceed that of colorectal cancer (10).

The risk for OC among these patients also varies according to the mutation variant. The highest risk has been reported in patients with MSH6 mutation, approximately 33% (14).

Understanding these genetic and epidemiological factors is crucial for developing targeted screening and prevention strategies for gynecologic malignancies among LS patients. Early identification and intervention can significantly improve outcomes for these high-risk individuals.

## 3 Screening

Screening for LS in the general population has gained prominence due to its potential to identify individuals at risk for hereditary malignancies. Early identification of MMR mutation carriers is crucial, as enhanced surveillance through regular colonoscopy and preventive measures has been shown to effectively reduce the morbidity and mortality associated with the syndrome (15).

### 3.1 Current guidelines for screening and risk assessment

Screening for LS in the general population begins with a thorough assessment of family and personal medical histories. Both the American College of Obstetricians and Gynecologists (ACOG) (16) and the NCCN (5) recommend evaluating histories of colorectal, endometrial, and other associated cancers to identify those at risk for LS. Healthcare providers are advised to assess the number of affected relatives and their ages at diagnosis to determine individuals who may require further investigation (5, 16).

Patients with a significant family history of colorectal or endometrial cancer (EC) should be referred for genetic counseling and testing (5, 16). Genetic counselors play a crucial role in educating patients about the inheritance patterns of LS, the implications of genetic testing, and potential outcomes. If a pathogenic variant is identified, family members may be offered cascade testing to determine their risk and consider preventive measures (16, 17).

### 3.2 Selective vs. universal screening approaches

Selective screening for LS involves testing individuals with high risk features according to the Amsterdam or Bethesda criteria (18). Despite its advantages, research suggests that this approach may miss a significant number of cases due to incomplete clinical data or the complexity of diagnostic guidelines (19). In contrast, universal screening—where all patients newly diagnosed with colorectal or EC are tested—has been shown to be more effective, leading to earlier detection and improved clinical outcomes (16). Consequently, several guidelines, including those from the National Institute for Health and Care Excellence (NICE) (20), the NCCN (5), and the joint recommendations by the European Society of Gynaecological Oncology (ESGO), the European Society for Radiotherapy and Oncology (ESTRO), and the European Society of Pathology (ESP) (21), advocate for LS testing in all patients diagnosed with EC.

The Mallorca Group also endorses these recommendations, particularly emphasizing that screening programs should focus on patients diagnosed with EC, especially those under the age of 70 (19). The Manchester International Consensus Group (MICG) (22) further recommends universal screening for LS in women diagnosed with EC, provided the necessary resources are available. The MICG specifically emphasizes the importance of screening for LS in women diagnosed with EC at or before the age of 60. Additionally, screening is recommended for women of any age with a personal history of metachronous or synchronous cancers associated with LS, those with a first-degree relative diagnosed with an LS-related cancer at or before the age of 60, or women whose pathological features suggest an LS-associated cancer.

### 3.3 Screening for LS in ovarian cancer

For OC, the MICG recommends screening for LS in women ≤50 years of age and in women of any age with epithelial non-serous and non-mucinous histology (22). This approach reflects evolving insights into LS-associated malignancies and highlights the importance of extending screening to other cancer types beyond CRC and EC when risk factors are present.

### 3.4 Screening protocols

The diagnosis of LS follows a multi-step approach aimed at identifying tumors with MMR deficiencies and confirming the presence of pathogenic genetic mutations. Current guidelines from leading organizations all highlight the critical role of immunohistochemistry (IHC) in identifying MMR deficiencies and guiding subsequent genetic testing.

According to the NICE guidelines, the initial diagnostic step involves the use of IHC to identify tumors with MMR deficiency by staining for the four MMR proteins—MLH1, MSH2, MSH6, and PMS2 (19). A normal IHC result indicates that all four proteins are normally expressed, suggesting the absence of MMR deficiencies. In contrast, the loss of expression of one or more of these proteins suggests an MMR defect and directs further genetic testing. The IHC pattern can pinpoint which gene may be mutated, as the loss of expression of certain proteins suggests specific gene defects or defects in their associated protein dimers:

- Loss of MLH1 or both MLH1 and PMS2: When this pattern is observed, MLH1 promoter hypermethylation testing of tumor DNA is recommended. If hypermethylation is absent, this indicates that germline genetic testing should be performed to confirm LS.
- Loss of MSH2, MSH6, or isolated PMS2: In these cases, germline genetic testing is directly recommended without further tumor testing (19).

The MICG (22) also supports IHC as the first diagnostic test for LS. When IHC results show a loss of MMR protein, further testing is recommended. If MLH1 loss is observed, promoter methylation-specific PCR should be performed to assess whether the loss of MLH1 expression is due to promoter hypermethylation, which would indicate a sporadic case rather than hereditary LS. In the absence of hypermethylation, germline genetic testing is pursued to confirm the diagnosis. In addition, the group states that MSI polymerase chain reaction (PCR) testing may also be a useful alternative to IHC, but requires non-neoplastic tissue, which can make the process more labor-intensive.

As opposed to IHC which provides information on the specific MMR genes affected, MSI testing primarily indicates overall instability (17). Several disadvantages in MSI testing, such as variations in different tumor regions (23), rates of false negative results (24) and variability in MSI markers used in different centers (25), favor the use of IHC as the primary screening method.

The NCCN guidelines similarly recommend the use of IHC and/or MSI testing to identify individuals at higher risk for LS. If abnormalities are found through tumor screening, germline testing of the MMR genes should be offered. This includes the four core MMR genes as well as EPCAM, which may also harbor mutations in some cases (5).

The ESGO/ESTRO/ESP guidelines endorse MMR IHC as the preferred method for evaluating MMR status. If MLH1 and PMS2 loss is detected, testing for MLH1 promoter methylation is recommended to differentiate sporadic cases from hereditary ones. While MSI testing through PCR methods is an alternative, it is considered more laborious and less informative regarding which specific MMR genes are affected (21).

The guidelines suggest that the combination of IHC testing followed by MLH1 promoter testing is likely to be the most cost-effective approach for identifying Lynch syndrome in patients with endometrial cancer (20, 21).

In summary, IHC is consistently recommended across all guidelines as the first diagnostic step for LS. Loss of MMR protein expression on IHC guides further testing, including MLH1 promoter methylation testing or direct germline testing, depending on which protein(s) are lost. MSI testing remains an alternative to IHC but is more labor-intensive and provides less specific information.

## 4 Preventive measure for gynecological malignancies

Prevention of gynecological malignancies in patients with LS requires a comprehensive approach that emphasizes patient education, regular surveillance, and the adoption of preventive measures.

### 4.1 Patient education

Patient education is vital in managing LS and its associated cancer risks, particularly for EC. Empowering patients through education supports informed decision-making, enhances self-efficacy, and improves the effectiveness of screening (26). Guidelines from ACOG and the MICG emphasize personalized consultations addressing LS risks, surveillance, fertility considerations, and preventive strategies, including surgery and pharmaceutical options (16, 22).

The Mallorca Group highlights the role of genetic counseling in understanding cancer risks, interpreting test results, and guiding at-risk relatives, while educating patients on cancer symptoms ensures early detection and better outcomes (19).

### 4.2 Surveillance for gynecologic malignancies associated with Lynch syndrome

#### 4.2.1 Surveillance for EC

This includes a range of recommendations from several leading medical organizations, each aimed at early detection and patient education (**Table 1**). The ACOG recommends that women with LS should be informed of their increased risk of EC. Starting at age 30–35, annual endometrial biopsy or transvaginal ultrasound may be considered to screen for early signs of malignancy (16).

**Table 1:** Recommendations for endometrial cancer surveillance in LS patients.

Committee	Recommendation
**ACOG** (16)	Annual endometrial biopsy or TVUS, starting at age 30–35.
**NICE** (20)	Focus on awareness of early gynecological cancer symptoms.
**Mallorca Group** (17)	Annual endometrial biopsy or TVUS starting at age 30–35.
**NCCN** (5)	Every 1-2 years endometrial biopsy, starting at age 30–35
**MICG** (22)	Emphasize symptom awareness Annual reviews from age 25.
**ESGO/ESTRO/ESP** (21)	Annual TVUS and endometrial biopsy MSH2 carriers: from age 30 MLH1 carriers: from age 35 MSH6 carriers: from age 40

The NICE guidelines take a slightly different approach, focusing on the importance of raising awareness of early gynecological cancer symptoms as a potential strategy for improving early diagnosis. While the effectiveness of formal gynecological surveillance in reducing cancer incidence or severity remains uncertain, it is included in cost-effectiveness models, underscoring its possible utility in LS patients (20).

According to the Mallorca Group, endometrial sampling or transvaginal ultrasound may be initiated for LS patients starting at age 30–35 (17). Similarly, the NCCN (5) guidelines suggest screening with endometrial biopsy every 1–2 years from age 30–35. However, transvaginal ultrasound is not recommended as a primary screening tool in premenopausal patients due to the variability of endometrial thickness during the menstrual cycle. In postmenopausal patients, while it may be considered, its sensitivity and specificity are not sufficient to support a formal recommendation.

The MICG takes a more conservative stance, advising against routine invasive gynecological surveillance for carriers of LS -pathogenic variants, citing insufficient evidence that it improves outcomes compared to symptom awareness and prompt investigation of “red flag” symptoms. These symptoms include abnormal bleeding, weight loss, bloating, changes in bowel habits, recurrent urinary symptoms, and abdominal pain. The Group suggests annual reviews starting at age 25, focusing on patient education regarding these symptoms rather than invasive screening (22).

Finally, the ESGO/ESTRO/ESP guidelines recommend a gene-specific approach to surveillance, beginning annual transvaginal ultrasound and endometrial biopsy at age 30 for MSH2 carriers, at age 35 for MLH1 carriers, and at age 40 for MSH6 carriers. This surveillance is advised until patients undergo a prophylactic hysterectomy, a definitive risk-reducing measure (21).

These surveillance protocols, while varied, underscore the importance of a personalized approach to care in women with LS, integrating patient education, symptom awareness, and in some cases, routine screening or preventive surgery.

#### 4.2.2 Surveillance for OC

Surveillance for OC in patients with LS is not routinely recommended due to the lack of evidence supporting its effectiveness in reducing mortality. Recent trials such as the UK Collaborative Trial of Ovarian Cancer Screening (UKCTOCS) (27) and The Prostate, Lung, Colorectal and Ovarian (PLCO) (28), failed to show survival benefit in patients screened for ovarian cancer.

Based on these findings, the ACOG specifically advises against routine OC screening for women with LS (16). This aligns with the Mallorca Group, which also concludes that there is no proven benefit to OC screening in this population (17).

The NCCN guidelines further emphasize that available data do not support routine OC screening for LS patients. While screening with CA-125 levels and pelvic ultrasound may be considered in certain contexts, such as preoperative planning, these tools are not endorsed for routine cancer surveillance due to their limited sensitivity and specificity in detecting early-stage disease (5).

### 4.3 Risk reducing surgery

Risk-reducing surgeries play a crucial role in managing the elevated risk of gynecological cancers in women with LS, particularly for those who have completed childbearing. The ACOG recommends that women consider undergoing risk-reducing bilateral salpingo-oophorectomy (BSO) after completing childbearing, typically between the ages of 35 and 40. This procedure significantly reduces the risk of OC (16).

The NICE guidelines similarly recommend that women with LS discuss the option of risk-reducing surgeries, including hysterectomy and BSO, after childbearing is completed. This ensures that patients are fully informed about the potential benefits and timing of these procedures (20).

The MICG also supports risk-reducing surgeries, advising that women at high risk for gynecological cancers, such as those with LS, consider procedures like hysterectomy and BSO. Annual discussions are recommended to assess the timing of these surgeries, with a general suggestion to offer them between the ages of 35 and 40 (22).

The Mallorca Group recommends that risk-reducing surgery, including hysterectomy and BSO, be considered after childbearing is complete, typically between the ages of 40 and 45, reflecting a slightly later window for intervention (17).

According to the NCCN guidelines, while total hysterectomy has not been conclusively shown to reduce endometrial cancer mortality, it does reduce the incidence of the disease and is considered a valid risk-reducing option. The timing of hysterectomy can be individualized based on the patient’s reproductive status, comorbidities, family history, and the specific LS gene involved, as EC risk varies by gene mutation. For patients undergoing colorectal surgeries, such as those related to colorectal cancer resection, coordinating hysterectomy with these procedures may be beneficial. Risk-reducing hysterectomy with BSO is typically considered starting at age 40, while delaying BSO until age 50 may be an option. Additionally, salpingectomy may reduce OC risk and is a consideration for premenopausal women not yet ready for oophorectomy (5).

The ESGO/ESTRO/ESP guidelines advocate for risk-reducing hysterectomy and BSO after childbearing is complete and, preferably, before the age of 40. These surgeries significantly reduce the risk of both endometrial and ovarian cancers in women with LS (**Table 2**).

**Table 2:** Risk-reducing surgeries guidelines.

Guideline	Type of Risk-Reducing Surgery	Advised Age
**ACOG** (16)	Bilateral salpingo-oophorectomy (BSO)	After childbearing, typically age 35–40
**NICE** (20)	Hysterectomy and oophorectomy	After childbearing is complete
**MICG** (22)	Hysterectomy and BSO	No earlier than age 35–40, reviewed annually
**Mallorca Group** (17)	Hysterectomy and BSO	After childbearing, typically age 40–45
**NCCN** (5)	Hysterectomy and BSO; delayed BSO possible; salpingectomy as an option for premenopausal women not ready for oophorectomy	Hysterectomy at age 40, delayed BSO at age 50
**ESGO/ESTRO/ESP** (21)	Hysterectomy and BSO	After childbearing, preferably before age 40

Despite the benefits of risk reducing surgery in patients with LS, premenopausal women undergoing BSO should be offered estrogen replacement therapy to alleviate menopausal symptoms and support bone health (15, 19).

### 4.4 Chemoprevention

While prophylactic surgeries remain the most definitive means of risk reduction, chemopreventive strategies, such as the use of OCPs and progesterone-based treatments, have garnered significant attention for their potential to lower cancer incidence in women with LS.

Observational studies in the general population have demonstrated that progestin-containing OCPs reduce incidence by approximately 50%, which provides a strong rationale for their use in women with LS (29)​. A pivotal randomized, multicenter study by Lu et al. (30) evaluated the short-term effects of progestin-containing OCs and depo-medroxyprogesterone acetate (DMPA) in women with LS. The study demonstrated that both treatments induced significant reductions in endometrial epithelial proliferation, a key marker of cancer risk. Over three months, participants treated with either OCPs or DMPA exhibited decreased endometrial proliferation and histological changes consistent with progestin action, supporting the theory that hormonal suppression of the endometrium could serve as an effective chemoprevention strategy in LS.

In addition, Dashi et al. (29), provided evidence from a retrospective cohort study of 1128 women with MMR gene mutations, showing that OCPs use for at least one year was associated with a significantly reduced risk of EC (HR 0.39, 95% CI 0.23–0.64). The study also found that this chemopreventive effect was consistent with data from the general population, suggesting that hormonal modulation through OCPs could be a viable, non-invasive option for risk reduction in LS​.

These studies provide support for the use of OCPs and progesterone-based treatments in women with LS, thus making them an acceptable option for patients with LS who are not ready to pursue risk-reducing surgery. Both the NCCN (5) and the MICG (22) recommends that the combined OCPs is considered for women at risk of LS.

### 4.5 Lifestyle modifications

Lifestyle interventions, particularly those aimed at addressing obesity, physical activity, and dietary habits, play a significant role in reducing the risk of EC (31, 32). Obesity is one of the strongest modifiable risk factors, with evidence showing that obese women have significantly higher risks compared to women with a normal body mass index (BMI). Weight loss through behavioral interventions such as diet and exercise, and more extreme measures like bariatric surgery, have demonstrated potential in reducing endometrial cancer risk markers, including insulin resistance and hormone levels. Physical activity is also linked to a reduced risk, with moderate to high-intensity exercise offering up to a 30% reduction in risk, even when factoring in BMI (33). As a result, the MICG advises that women diagnosed with LS maintain a healthy diet, avoid obesity, engage in regular exercise, avoid smoking and avoid known carcinogens as part of their prevention program (22).

## 5 Management of endometrial cancer in patients with Lynch syndrome

**Figure 1:**
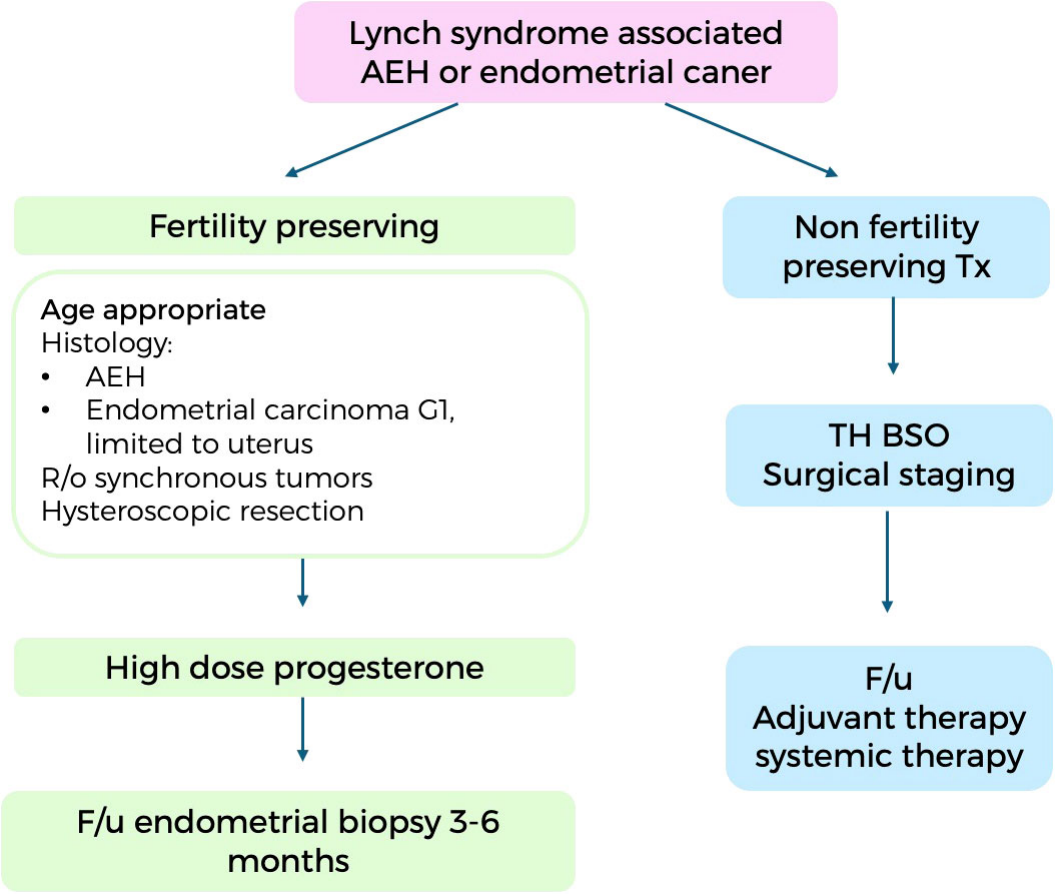
Suggested algorithm for the management of hyperplasia with atypia and grade 1 endometrial cancer in patients with Lynch syndrome. AEH, atypical endometrial hyperplasia; Tx, treatment; TH, total hysterectomy; BSO, bilateral salpingoophorectomy; F/u, follow up; R/o, rule out.

When EC is diagnosed in a LS patient, the treatment approach is generally similar to that for sporadic cases. Surgical management including total hysterectomy with BSO and surgical staging remains the standard of care (31). However, there are some important requiring specific considerations when treating these patients (**Figure 1**).

### 5.1 Fertility preserving treatment

Fertility-preserving treatment in patients with atypical endometrial hyperplasia (AEH) or early-stage EC is a critical consideration, particularly in young women who desire future pregnancies.

According to the NCCN (35), British Gynecology Cancer Society (BCGS) (36) and ESGO guidelines (21), fertility-preserving treatment may be considered for women with having a strong desire for future child bearing and meeting the following criteria:

1. Histologic confirmation of disease: This includes a confirmed diagnosis of grade 1, endometrioid adenocarcinoma limited to the endometrium (Stage IA), or AEH, based on endometrial (21, 35).
2. Absence of myometrial invasion or metastasis: Imaging studies (MRI preferred) should confirm that the disease is confined to the endometrium, with no evidence of myometrial invasion, cervical involvement, or distant metastasis (34, 36).
3. Patients should have no contraindications to hormonal therapy: This includes evaluating the patient’s medical history for contraindications to progestins or other hormonal treatments (35).
4. Strong patient commitment to follow-up: Patients need to be highly compliant with close follow-up, as fertility-preserving treatment requires ongoing monitoring of the disease (35).

The cornerstone of fertility-preserving treatment in both AEH and early-stage EC is hormonal therapy. The most commonly recommended regimen across all guidelines involves high-dose progestin aimed at inducing regression of the hyperplasia or cancer. The common regimens include megestrol acetate and levonorgestrel-releasing intrauterine system (37). The duration of hormonal treatment varies but typically extends for 6–12 months, with regular follow-up biopsies every 3–6 months to assess for disease regression (21, 35).

Once a complete response is achieved and fertility-preserving treatment is concluded, patients who wish to conceive are encouraged to attempt pregnancy promptly, either naturally or through assisted reproductive technologies. Post-pregnancy, definitive surgical management is recommended to minimize the risk of recurrence (21, 35).

The response among patients with AEH and early stage EC varies across studies. A comprehensive review indicates that the complete response rate ranges from 25% to 89% (38). A meta-analysis including 408 patients reported pooled complete response rate of 76.2% (39). In another cohort, after six months of treatment, 89.3% of patients achieved complete regression (40). Despite these promising results, reported recurrence rates ranged from 19.2% to 33.8% (38), thus underscoring the need for careful patient selection and monitoring.

EC in patients with LS commonly presents at younger age as compared with sporadic cases, thus patients may often present at reproductive age (1). This requires considerations for fertility preserving approach.

Fertility-preserving treatment in patients with LS has shown clinical response rates ranging from 66% to 76.3%. However, these initial favorable outcomes are tempered by high recurrence rates, which can vary significantly, with reports ranging from 20.1% to as high as 100% (41). A recent study from Italy, evaluated the effect of molecular classification of endometrial cancer on fertility sparing treatment. The study concluded that patients with miss-match repair deficiency (dMMR), as present in LS, had lower response to progestins as well as the highest recurrence rate, when compared to other molecular sub-classes (42). Thus, LS-associated cancers, may present a more aggressive disease compared to sporadic cases. The prognosis for those undergoing fertility-sparing treatment is influenced by factors such as the presence of synchronous tumors and the specific genetic mutations related to LS. A substantial number of patients may present with synchronous cancers, especially OC and colorectal cancers, further complicating treatment and requiring vigilant, ongoing surveillance. Studies have shown that women with LS who receive conservative treatment for AEH or early-stage EC face high rates of relapse, with recurrence occurring at 12, 18, or 24 months after initial complete response (43).

The European Society of Gynecological Oncology (ESGO)/European Society of Human Reproduction and Embryology (ESHRE)/and the European Society for Gynecological Endoscopy (ESGE) guidelines approach this issue of fertility-sparing treatment in women with LS with AEH or early stage EC (43):

1. Patient selection: Specific factors must be evaluated, including the patient’s age at diagnosis and the potential for disease progression, as women with LS may have a younger age of diagnosis and a higher risk of aggressive disease.
2. Risks of Recurrence and Response: Women with LS may experience higher rates of resistance to conservative treatment and recurrences. The guidelines suggest that hysteroscopic resection may improve outcomes in these cases. The recurrence rates and response to treatment can vary significantly, and careful monitoring is essential.
3. Prognosis: The prognosis for women with LS who undergo fertility-sparing treatment can be complex. While outcomes can be favorable, the presence of LS is associated with an increased risk of synchronous OC and other malignancies, which complicates the overall management and prognosis (44).

### 5.2 Systemic treatment

Systemic therapy is essential in managing advanced or recurrent endometrial cancer (EC). For many years, treatment centered on chemotherapy, primarily a combination of carboplatin and paclitaxel (45). However, the incorporation of molecular tumor profiling has transformed treatment approaches for these patients (46). Endometrial cancers associated with Lynch syndrome (LS) are characterized by high microsatellite instability (MSI-H), which influences treatment decisions and makes these tumors particularly responsive to immunotherapy (47).

The primary immunotherapy agents approved for treating mismatch repair-deficient (dMMR) patients with advanced or recurrent EC are programmed death (PD)-1 inhibitors, which have demonstrated efficacy in this population.

The GARNET trial (48), evaluated the efficacy of dostarlimab, a humanized anti-PD-1 monoclonal antibody, in dMMR EC patients. Results indicated an objective response rate (ORR) of 42%, suggesting substantial anti-tumor activity. Dostarlimab was shown to have a favorable safety profile, with sustained responses over time, indicating its potential as a viable option for dMMR patients. Subsequently, the phase 3, RUBY trial (49), assessed dostarlimab in combination with carboplatin and paclitaxel compared to a placebo with the same chemotherapy regimen. In the dMMR subgroup, the dostarlimab combination significantly improved progression-free survival (PFS) [64% in the dostralimab group compared to 15% in the placebo group, hazard ratio (HR) for disease progression or death was 0.28]. In addition, benefit in 24 months overall survival (OS) was also shown in this population (83.3% with dostarlimab and 58.7% with placebo, HR 0.3).

Pembrolizumab, another PD-1 inhibitor, is approved for dMMR/MSI-H advanced or recurrent EC. The KEYNOTE-158 study (50), evaluated its effectiveness across 27 tumor types, with an ORR of 34.3% in patients with MSI-H tumors. In a focused analysis on EC patients by O’Malley et al. (51), an ORR of 48% was achieved, with a median PFS of 13.1 months and an acceptable toxicity profile. Additionally, the NRG-GY018 phase 3 trial evaluated pembrolizumab combined with carboplatin and paclitaxel, followed by pembrolizumab maintenance. The PFS HR in the dMMR group was 0.30, underscoring a significant benefit for this combination therapy (52).

In response to emerging evidence, the NCCN has recently added the triplet regimens of pembrolizumab with carboplatin/paclitaxel and dostarlimab with carboplatin/paclitaxel as preferred primary therapy options for patients with stage III or IV disease (36). This endorsement reflects the growing role of immunotherapy in the management of LS-associated and dMMR/MSI-H EC.

## 6 Conclusion

Managing gynecologic cancers risk in LS patients requires a nuanced approach integrating genetic testing, personalized surveillance, and preventive measures. Despite advances in diagnostic accuracy, screening protocols remain inconsistent, and evidence on their impact varies. Risk-reducing surgeries, especially for women past childbearing, continue to be highly effective, while hormonal and chemopreventive strategies offer non-invasive options for selected patients. Systemic therapies, particularly immunotherapies, are demonstrating significant promise for advanced cases, improving overall and progression-free survival. Future research should aim to refine screening guidelines and expand options for early intervention to improve outcomes for this high-risk group.
